# P-2178. Can serum ferritin be used as a marker of severity in dengue fever?: a prospective observational study from North India

**DOI:** 10.1093/ofid/ofae631.2332

**Published:** 2025-01-29

**Authors:** Sayan Maharatna, Sudip Kumar Datta, Ranveer Singh Jadon, Manish Soneja, Dr Ashutosh Biswas, Lalit Dar, Naveet Wig

**Affiliations:** All India Institute of Medical Sciences, New Delhi, New Delhi, Delhi, India; All India Institute of Medical Sciences, New Delhi, New Delhi, Delhi, India; All India Institute of Medical Sciences, New Delhi, New Delhi, Delhi, India; All India Institute Of Medical Sciences, Delhi, Delhi, India; AIIMS Bhubaneswar, Bhubaneswar, Orissa, India; All India Institute of Medical Sciences, New Delhi, India, New Delhi, Delhi, India; All India Institute of Medical Sciences, DELHI, Delhi, India

## Abstract

**Background:**

Dengue has a variable clinical course and there is no standardised marker for predicting dengue severity. Studies have shown higher ferritin levels in dengue compared to other febrile illnesses. Prospective studies to show correlation of ferritin with dengue severity are lacking.

Demographic profile and workflow

**Figure:**
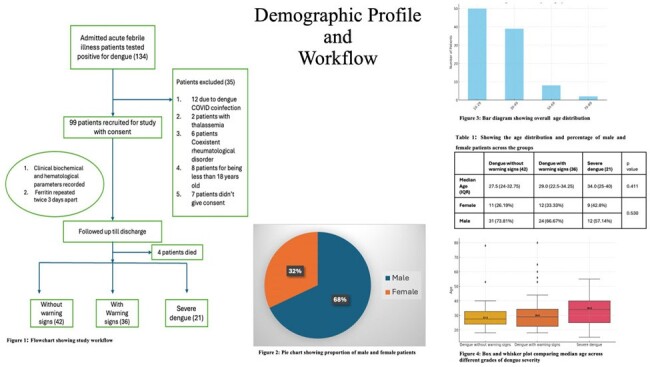

From 134 patients 99 were selected for the study , 35 were excluded as per the exclusion criteria (figure 1 flowchart). Majority of the patients were young (figure 3) with male predominance (figure 2), the male female percentages and age did not vary significantly across different grades of severity (table1 and figure 4).

**Methods:**

Single centre prospective observational study conducted over 2 years. Patients of chronic inflammatory diseases, thalassemia, haematological malignancy or severe anemia (Hb < 7gm/dl) were excluded. Two ferritin samples 3 days apart measured by automated BECKMAN COULTER DxI.800 immunoassay analyzer.

Clinical features and median ferritin value across different dengue severties

**Figure:**
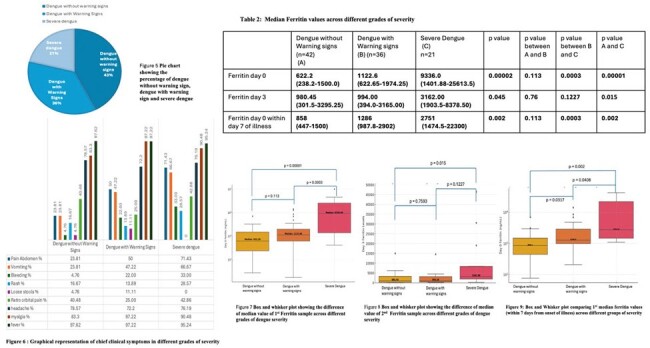

In Figure 5 the pie chart shows that majority of the patients were dengue without warning signs , approximately 1/5th were severe dengue. Figure 6 shows that initial clinical features were more or less similar in the three groups of severities , however bleeding, pain abdomen , vomiting were more common in severe dengue group. Table 2 showing median ferritin values of day 0 and day 3 ferritin in different severity groups and whether their differences are statistically significant between each groups or not. The Box and whisker plots in figure 7, 8 and 9 shows the same with corresponding p values and Median ferritin level annotated.

**Results:**

Total study population was 99 patients with median age of 28 years (24.5-34.5) and male predominance (68%). 2009 WHO classification wise 43% were dengue without warning signs 36% dengue with warning signs and 21% were severe dengue patients. Both day 0 and day 3 ferritin median value were higher across all three groups and the difference were statistically significant (p=0.00002 and p=0.045 respectively). Day 0 ferritin median value difference was statistically significant between severe dengue and dengue without warning sign (0.00001) and warning sign group (p=0.0003). In case of early ferritin sample (within day 7 of illness) it was significantly different between all groups. Serum ferritin also showed strong positive correlation with AST, ALT and negative correlation with Albumin levels. ROC curve analysis showed that a ferritin cut off of 9185 ng/ml with AUC 0.811, standard error 0.06 and 95% CI of 0.69-0.92 could differentiate severe from non severe dengue with 52.4% sensitivity and 97.4% specificity. For early ferritin sample cut off becomes 2132 ng/ml with a 70% sensitivity and 82.4% specificity with AUC 0.821, standard error 0.085 and 95% CI of 0.65-0.98. Multivariate logistic regression shows baseline platelet counts < 50000 cells/uL [OR 4.7, p=0.02, 95% CI 1.19-18.54] and Albumin < 3.5 gm/dL [OR 6.33 p=0.007 95% CI 1.67-24.06] to be independent predictors of severe dengue. Day 0 ferritin level > 9185ng/mL gave an Odd’s ratio of 10.47 with p=0.052 and 95% CI [0.98-112].

Correlation of ferritin with various biochemical parameters and Ferritin cutoff value to differentiate Severe from Non severe dengue by ROC curve

**Figure:**
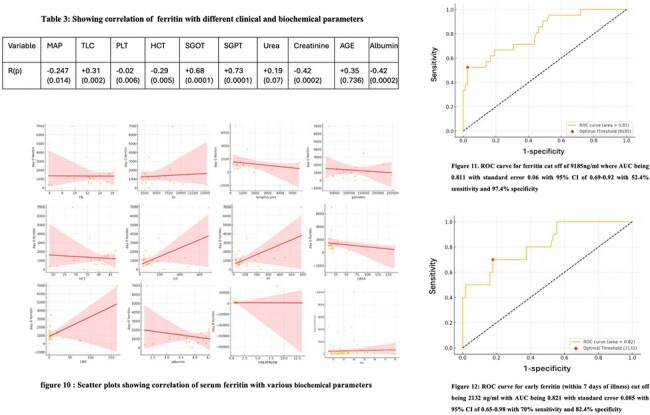

Table 3 and Figure 10 Scatter plot showing strong positive correlation between serum ferritin and SGOT/ SGPT levels, and negative correlation with serum albumin level .Figure 11 ROC curve analysis showing the ferritin cutoff being 9185ng/ml to differentiate between severe and non severe dengue. If sample taken within day 7 of illness the cut off becomes 2132 ng/ml (Figure 12)

**Conclusion:**

Ferritin can be a useful biomarker for differentiating different grades of dengue severity especially if sample taken in the 1st week of illness. Serum ferritin cut off values can be used to predict severe dengue.

Multivariate Logistic regression to find independant predictor of severe Dengue

**Figure:**
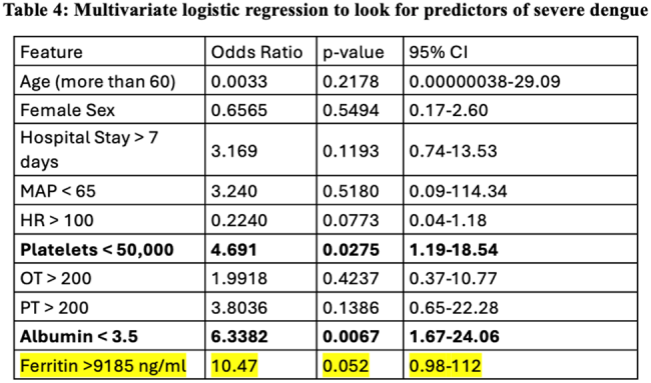

Table 4 showing Multivariate logistic regression result, which reveals baseline platelet count <50000 and Albumin level <3.5gm/dL to be independent predictor of severe dengue.Day 0 Ferritin cutoff of 9185 ng/dL as per the ROC curve analysis shows that the Odds of prediction of severe dengue is 10.47 (p=0.052 and 95% CI 0.98-112).

**Disclosures:**

All Authors: No reported disclosures

